# A Case of an Incidentally Removed Lingual Osseous Choristoma

**DOI:** 10.1155/2020/3498915

**Published:** 2020-03-17

**Authors:** Tomotaka Hemmi, Jun Suzuki, Satoko Sato, Masumi Tabata, Kojiro Watanabe, Mitsuru Sugawara, Yukio Katori

**Affiliations:** ^1^Department of Otolaryngology, Tohoku Kosai Hospital, 2-3-11 Kokubun-cho, Aoba-Ku, Sendai 980-0803, Japan; ^2^Department of Otolaryngology, Head and Neck Surgery, Tohoku University School of Medicine, 1-1 Seiryo-machi, Aoba-Ku, Sendai 980-8574, Japan; ^3^Department of Pathology, Tohoku University Hospital, 1-1 Seiryo-machi, Aoba-Ku, Sendai 980-8574, Japan; ^4^Department of Otolaryngology, Osaki Citizen Hospital, 3-8-1 Furukawahonami, Osaki, Miyagi 989-6136, Japan

## Abstract

Lingual osseous choristoma is a rare benign tumor consisting of normal matured bone tissue. It was first reported in 1913, and less than 100 cases of lingual osseous choristomas, mainly in their twenties and thirties, have been reported in the English literature until now. Here, we report an additional case of lingual osseous choristoma, in an elderly patient, that was incidentally removed by coughing and cured without additional interventions. An 89-year-old male patient was referred to our department for an evaluation of chronic cough. When we examined his oral cavity and pharynx, he expectorated a 10 -mm mass which was histologically diagnosed as an osseous choristoma. We confirmed the well-defined, rounded, high-density mass with a tiny pedicle on the base of the tongue in previous cervical spine CT images. No signs of recurrence were found during the 15-month follow-up examination. Our case serves as a reminder of this rare entity in the diagnosis of tongue masses of the elderly.

## 1. Introduction

The term choristoma was coined to define a tumor-like lesion composed of normal tissue in an abnormal region [1]. Subsequently, a bone-forming tumor of the tongue was first reported by Monserrat in 1913, and the author termed it “lingual osteoma” [2]. Choristomas may arise from osseous tissue, cartilaginous tissue, lingual thyroid, salivary gland, glial tissue, gastric mucosal tissues, and other tissues, and the term of “osseous choristoma” was used to define soft tissue osteomas in the head and neck by Krolls et al. [3]. Although the etiopathology of osseous choristoma is still unknown because of its rarity, development theory and posttraumatic (reactive) theory are widely recognized as its pathogenesis [4]. Here, we present a case of osseous choristoma of the tongue that is the oldest case known to date, and the first case of incidental removal by coughing and cured without additional treatments.

## 2. Case Report

An 89-year-old male was referred to our department for an evaluation of prolonged cough from a physician after screening tests. The patient's past medical history revealed presence of hypertension, subdural hematoma, and lumber canal stenosis. The patient had no history of intraoral trauma.

**Figure 1:**
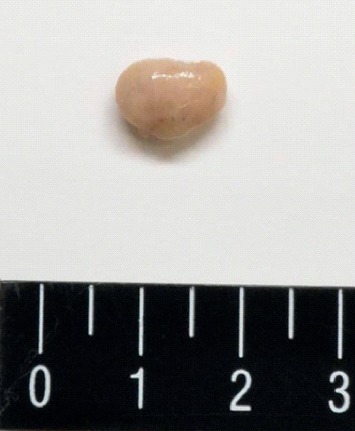
Gross specimen image. The specimen was sized at approximately 10 mm in diameter.

**Figure 2:**
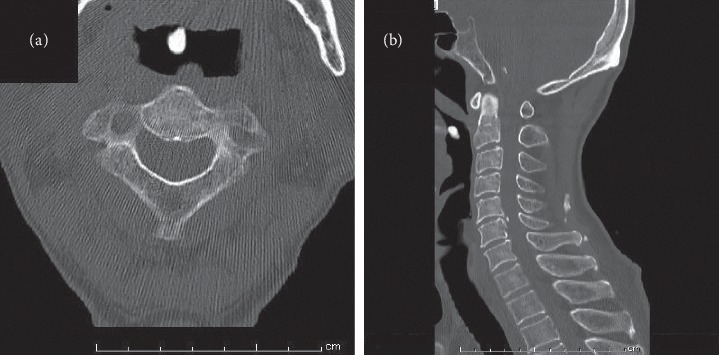
Computed tomography scan showing a well-defined, rounded, high-density mass with a tiny pedicle on the base of the tongue: (a) axial and (b) saggital.

When we examined the patient's oral cavity and pharynx, the patient furiously coughed and expectorated a small mass. The mass was approximately 10 mm in diameter and was covered with normal mucosa (Figure 1). We only observed mild bleeding on the base of the tongue by fiberoptic examination, but there were no other abnormal findings in the patient's oral cavity, pharynx, and larynx. We confirmed the well-defined, rounded, high-density mass with a tiny pedicle on the base of the tongue in prior cervical spine computed tomography (CT) images taken by the orthopedic department 6 months before the patient's first visit to our department (Figure 2). His chronic cough persisted even after removal of the mass. According to the upper gastrointestinal endoscopy results, we concluded that the patient's cough was due to gastroesophageal reflux disease.

**Figure 3:**
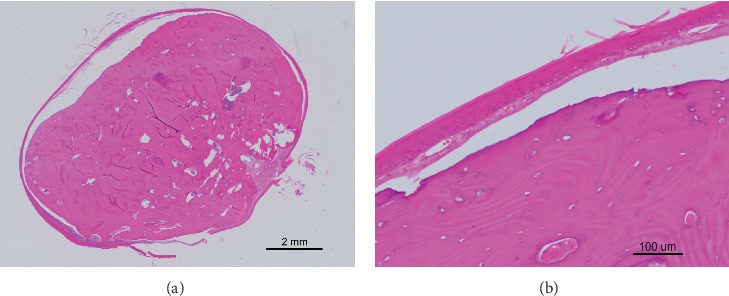
Histopathologic images. (a) Histologic examination shows well-circumscribed bone tissue (H&E ×20). (b) Mature bone is surrounded by fibrous connective tissue and covered with stratified squamous epithelium (H&E ×200).

Histological examination revealed that the mass consisted of circumscribed bone, and it was surrounded by benign-appearing stratified squamous epithelium (Figure 3). The bone tissue had lamellar structures. There was no evidence of malignancy in the specimen, and the histological diagnosis of an osseous choristoma was made. There were no signs of recurrence at the time of follow-up examination 15 months later. Written informed consent was obtained from this patient.

## 3. Discussion

Less than 100 cases of osseous choristomas of the oral and maxillofacial region have been reported in the English language literature, and the most common site is the tongue, especially the dorsal posterior third of the tongue near the foramen caecum and circumvallate papillae [5]. Gorini et al. provided a comprehensive review of 67 cases of the lingual osseous choristomas [6], and eight additional cases of osseous choristoma of the tongue were reported since their review [5, 7–12]. Including newly reported cases and our case, the patients' age ranged from five to eighty-nine years (mean: 28.9), with majority of the patients being in the second or third decades of life, and a strong female predilection (male : female, 19 : 50) was observed. To our knowledge, this is the oldest patient diagnosed with osseous choristoma. As such, we should consider osseous choristoma as a differential diagnosis of tongue masses in the elderly.

Osseous choristoma appears as a sessile or pedunculated mass usually covered by normal mucosa. The size of the lesion may vary from 3 to 50 mm at their largest diameter, and most osseous choristomas of the tongue are frequently asymptomatic [6]. The most frequent symptom is a lump, and patients rarely complain of dysphagia, a gagging sensation, pain, vomiting reflex, and nausea [6]. Although some authors have suggested that these symptoms are correlated with lesion size and location [2, 6], a comprehensive review of 58 cases of osseous choristomas reported no relationship between lesion size and symptoms [13]. In our case, the patient's chief complaint was prolonged cough, which we believe was not related to the lesion. A CT scan was performed 6 months before the patient's first visit to our department when the patient was asymptomatic.

The clinical differential diagnoses include tumor-like lesions, benign tumors, and malignant tumors [2, 14]. We must consider the location of the lesion to reach a correct diagnosis [6, 15]. Some authors suggest that CT scan is useful for the radiological diagnosis of lingual osseous choristomas [16]. On CT images, lingual osseous choristomas consists of well-defined, spheroid, bone-attenuation masses that arise from the base of the tongue [17, 18]. In this case, we observed the lesion in CT images taken for evaluation of the cervical spine. In particular, sagittal planes provided useful information such as continuity of the lesion with surrounding tissues. In some cases, magnetic resonance images (MRI) are also performed. We must pay attention to the fact that motion artifacts in the base of the tongue area easily occur in patients who have difficulty following instructions, e.g., children and elderly [19]. In our case, we were able to detect the lesion in the cervical spine CT images retrospectively, which reminds us that this rare entity could be found in cervical spine CT images.

The treatment of lingual osseous choristoma is surgical excision. Although recurrence of osseous choristomas of the masseter muscle has been reported [20], there have not been reports on the malignant evolution of lingual osseous choristomas thus far [7]. To our knowledge, our patient is the first reported case of a lingual osseous choristoma that was incidentally removed and cured without surgical interventions. As with our case, some lingual osseous choristomas with tiny pedicles might be removed without the patient's knowledge by coughing or swallowing. Adopting a “wait and see” approach might be an option for asymptomatic lingual osseous choristoma cases with no strong indication for surgery.

In conclusion, we have reported the oldest case of lingual osseous choristoma known thus far. CT scans are useful for the radiological diagnosis of lingual osseous choristomas. The “watch and wait” approach could be an option for asymptomatic lesions because lingual osseous choristomas with tiny pedicles spontaneously resolve.
